# Supplementary figures and images for: Long-term effects of a three-component lifestyle intervention on emotional well-being in women with Polycystic Ovary Syndrome (PCOS): A secondary analysis of a randomized controlled trial
Source: PLoS One. 2020 Jun 1;15(6):e0233876. doi: 10.1371/journal.pone.0233876 (PMC7263605; doi:10.1371/journal.pone.0233876)

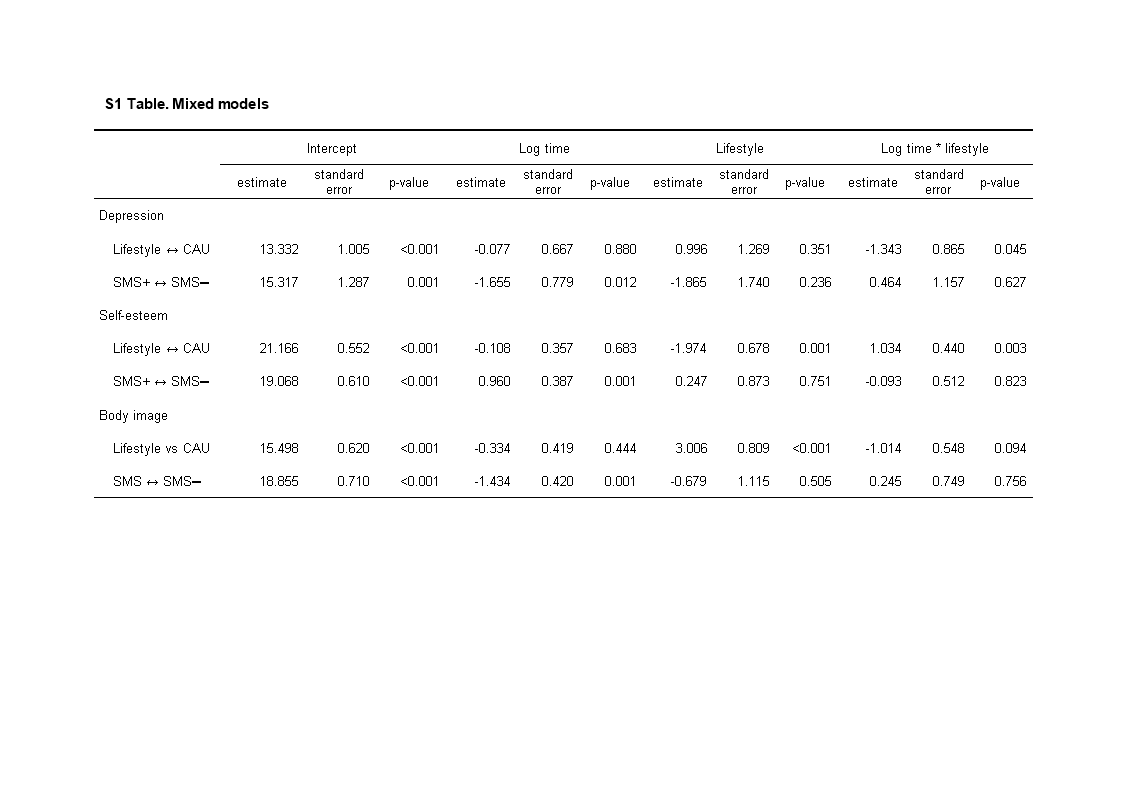

Supplement: S1 Table — (TIFF) [file pone.0233876.s001.tiff]

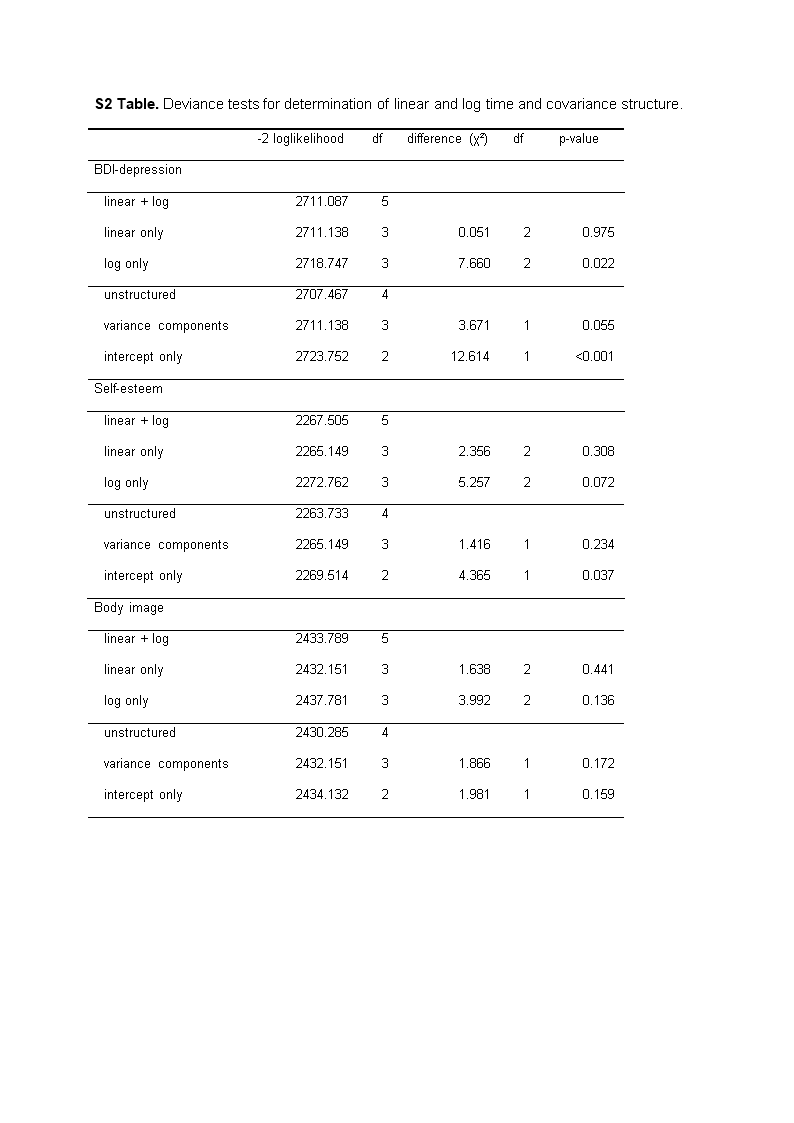

Supplement: S2 Table — (TIFF) [file pone.0233876.s002.tiff]
