# Supplementary material for: Long-term effects of a three-component lifestyle intervention on emotional well-being in women with Polycystic Ovary Syndrome (PCOS): A secondary analysis of a randomized controlled trial
Source: PLoS One. 2020 Jun 1;15(6):e0233876. doi: 10.1371/journal.pone.0233876 (PMC7263605; doi:10.1371/journal.pone.0233876)
Supplement: Attachment — Submitted filename: Rebuttal PlosOne.doc [file pone.0233876.s004.doc]

May 11^th^, 2020

Dear Dr. Atkin,

Thank you for reviewing our manuscript entitled ‘Long-term effects of a three-component lifestyle intervention on emotional well-being in women with Polycystic Ovary Syndrome (PCOS): a secondary analysis of a randomized controlled trial’.

We are grateful for the detailed and constructive comments received from the reviewers, as they will help us to improve the manuscript. You will find our specific responses to the questions and comments of the reviewers (*in italics)* and the corresponding textual changes made. In the revised manuscript, we have marked these textual changes in yellow.

With kind regards,

On behalf of all authors,

Geranne Jiskoot, MSc

Division of reproductive medicine, Department of Obstetrics and Gynecology, Erasmus MC

Wytemaweg 80, PO Box 2040, 3000 CA Rotterdam, the Netherlands

Phone: +31 (0) 6 44 184 834, E-mail: l.jiskoot@erasmusmc.nl

1. I feel spontaneous pregnancy during the study is an important information and it is worth mentioning it in the manuscript (it was included in the first submission but excluded in revised version).

*We included the information about spontaneous pregnancies in Table 1. The difference between LS without SMS compared to CAU was not significant (P=0.816), LS with SMS compared to CAU (P=0.198) and LS with SMS compared to LS without SMS (P=0.130).*

2. There has been difference in spontaneous pregnancy rates (during study) between the 3 groups. All women participating in the study wanted fertility, was spontaneous pregnancy during the study considered as a mediator/confounder? If not, could it be included?

*We agree with the reviewer that this could be an interesting possibility. Therefore, we have checked if spontaneous pregnancy mediated the effects of the lifestyle treatment on well-being scores. We found no mediation in the relationship between depression with spontaneous pregnancy (P=0.766), nor between self-esteem with spontaneous pregnancy (P=0.570) and body image with spontaneous pregnancy (P=0.890).*

3. In the baseline data table; the median weight was 84, 89, and 94.5kg in the control, LS, and LS+SMS groups respectively. While the median BMIs were 30.6, 33.5, and 33.5 kg/m2 respectively. I find it strange that the SL+SMS group was 5kg heavier than SL group but with similar BMI. Can this result be double checked?

*We double checked the data and included the SPSS output for weight, height and BMI below. The median weights as reported in the paper are correct. The LS with SMS is taller and heavier, therefore they have the same BMI as LS without SMS. We have added information about height in Table 1.*

| **Statistics** | | | | | |
| --- | --- | --- | --- | --- | --- |
| group | | | HEIGHT | WEIGHT | BMI |
| 1 Controle | N | Valid | 60 | 60 | 60 |
|  |  | Missing | 0 | 0 | 0 |
|  | Percentiles | 25 | 160.00 | 79.00 | 29.336 |
|  |  | 50 | 165.00 | 84.00 | 30.662 |
|  |  | 75 | 170.00 | 97.25 | 34.375 |
| 2 SMS- | N | Valid | 63 | 63 | 63 |
|  |  | Missing | 0 | 0 | 0 |
|  | Percentiles | 25 | 160.00 | 80.00 | 30.469 |
|  |  | 50 | 164.00 | 89.00 | 33.695 |
|  |  | 75 | 169.00 | 105.00 | 36.506 |
| 3 SMS+ | N | Valid | 60 | 60 | 60 |
|  |  | Missing | 0 | 0 | 0 |
|  | Percentiles | 25 | 161.25 | 85.25 | 31.227 |
|  |  | 50 | 167.00 | 94.50 | 33.577 |
|  |  | 75 | 170.00 | 105.75 | 37.071 |
